# Effect of ketogenic mediterranean diet with phytoextracts and low carbohydrates/high-protein meals on weight, cardiovascular risk factors, body composition and diet compliance in Italian council employees

**DOI:** 10.1186/1475-2891-10-112

**Published:** 2011-10-12

**Authors:** Antonio Paoli, Lorenzo Cenci, Keith A Grimaldi

**Affiliations:** 1 Department of Human Anatomy and Physiology, University of Padova, Padova, Italy; 2 Nutrition and Dietetic Service, San Bortolo Hospital, Vicenza, Italy; 3 Biomedical Engineering Laboratory, Institute of Communication and Computer Systems, National Technical University of Athens, Athens, Greece

## Abstract

**Background:**

There has been increased interest in recent years in very low carbohydrate ketogenic diets (VLCKD) that, even though they are much discussed and often opposed, have undoubtedly been shown to be effective, at least in the short to medium term, as a tool to tackle obesity, hyperlipidemia and some cardiovascular risk factors. For this reason the ketogenic diet represents an interesting option but unfortunately suffers from a low compliance. The aim of this pilot study is to ascertain the safety and effects of a modified ketogenic diet that utilizes ingredients which are low in carbohydrates but are formulated to simulate its aspect and taste and also contain phytoextracts to add beneficial effects of important vegetable components.

**Methods:**

The study group consisted of 106 Rome council employees with a body mass index of ≥ 25, age between 18 and 65 years (19 male and 87 female; mean age 48.49 ± 10.3). We investigated the effects of a modified ketogenic diet based on green vegetables, olive oil, fish and meat plus dishes composed of high quality protein and virtually zero carbohydrate but which mimic their taste, with the addition of some herbal extracts (KEMEPHY ketogenic Mediterranean with phytoextracts). Calories in the diet were unlimited. Measurements were taken before and after 6 weeks of diet.

**Results:**

There were no significant changes in BUN, ALT, AST, GGT and blood creatinine. We detected a significant (p < 0.0001) reduction in BMI (31.45 Kg/m^2 ^to 29.01 Kg/m^2^), body weight (86.15 kg to 79.43 Kg), percentage of fat mass (41.24% to 34.99%), waist circumference (106.56 cm to 97.10 cm), total cholesterol (204 mg/dl to 181 mg/dl), LDLc (150 mg/dl to 136 mg/dl), triglycerides (119 mg/dl to 93 mg/dl) and blood glucose (96 mg/dl to 91 mg/dl). There was a significant (p < 0.0001) increase in HDLc (46 mg/dl to 52 mg/dl).

**Conclusions:**

The KEMEPHY diet lead to weight reduction, improvements in cardiovascular risk markers, reduction in waist circumference and showed good compliance.

## Background

Obesity has become a health emergency in Western countries [1,2]. As is well known, obesity and in particular abdominal obesity is one of the principle risk factors for cardiovascular disease and along with dyslipidemia, hypertension and diabetes contributes to the metabolic syndrome [3]. Even though weight loss is a desired goal for most overweight individuals, and its health benefits have been clearly determined [4,5], there are still no definitive data on what dietary protocols are most effective in both the short and long term [6] or even what is the correct nutritional approach in general [7].

The most commonly accepted dietary strategy is based on relatively high levels of carbohydrates and low fat content but according to some studies these low fat diets yield only modest weight loss and suffer from low long-term compliance [8]. In fact adherence of overweight/obese individuals to high carbohydrate/low fat nutrition is often a problem because the majority have been shown to have dietary preferences for foods with a rich fat content. Furthermore rather than consume complex carbohydrates there is a tendency to prefer highly processed food containing simple sugars [9-11] such that a low fat diet can actually encourage the consumption of sugars and refined carbohydrates that can worsen weight problems and also facilitate dyslipidemia especially in insulin resistance individuals [12,13]. As a consequence of the relative inefficacy of these types of diet there has been increased interest in recent years in very low carbohydrate ketogenic diets (VLCKD) [14] that, have undoubtedly been shown to be effective, at least in the short to medium term, as a tool to tackle obesity, hyperlipidemia and some cardiovascular risk factors [15-18]. The Mediterranean diet is often proposed as the healthy standard but many of the advantages associated with it can actually be linked to life style and the true original Mediterranean diet did not contemplate the current high levels of refined carbohydrates on which the typical Italian diet is based [19,20]. For this reason the standard ketogenic diet is not associated with high compliance in populations, like the Italian, that are used to carbohydrate based diets. The objective of the present study, which was devised as a case pilot trial, is to assess the safety, compliance and effects of a "Mediterranean style" ketogenic diet that utilizes very low carbohydrate ingredients formulated to simulate the aspect and taste of common carbohydrate rich foods (e.g. pasta) and which contain phytoextracts intended to add beneficial effects of important vegetable components. The aim of using herbal extracts during the diet period was to reduce some commonly reported light side effects of ketogenic diets. The parameters measured include blood biomarkers, body composition, weight loss and compliance in a cohort of council employees in Rome, Italy.

## Methods

### Subjects

The pilot study group consisted of 106 Rome council employees (19 males and 87 female; mean age 48, 49 ± 10, 33). Inclusion criteria were: BMI ≥ 25, age > 18 years and < 65 years, currently on a carbohydrate rich diet (> 50% energy), [21] desire to lose weight and health status suitable for a modified ketogenic diet (Tisanoreica^®^) [22] i.e. normal renal function, not pregnant or lactating. After the start of the experimental protocol the subjects who began new exercise programs or pharmaceutical treatments would be excluded. A small rise in transaminase was not considered a condition for exclusion since mild alterations in GOT and GPT values are common in obese individuals. Subjects eligible for the study were invited to the IPA clinic (Istituto di Previdenza ed Assistenza - health services for public sector employees) to attend an orientation session. At the first visit it was explained that during the first three weeks it was necessary to almost totally exclude carbohydrates and a detailed menu containing permitted and non-permitted foods was provided to each participant, along with the components of the ketogenic Mediterranean with phytoextracts (KEMEPHY) diet described below. Anthropometric measures were performed and blood samples were taken from the subjects two-three days before and after they began the diet. Subjects received no monetary compensation for their participation and signed a voluntary consent form before initiating the diet. The ethical and clinical review committee of IPA and the European Nutrition Society approved the study protocol, informed consent form and information material provided to subjects.

### Diet

The KEMEPHY (ketogenic Mediterranean with phytoextracts) diet protocol was ketogenic during the first 3 weeks with approximately 34 g of CHO daily, using low carbohydrate high-protein meals and herbal teas [22] (Tisanoreica^® ^by Gianluca Mech SpA, Orgiano VI) (Table 1 and 2).

**Table 1:** diet composition in KEMEPHY (ketogenic Mediterranean with phytoextracts) diet.

	***KEMEPHY Week 1-3***	***KEMEPHY Week 4-6***
**Energy Kcal**	**1098 ± 21.3**	**1186 ± 107**

**Protein, g/day** **(% daily Energy)**	99 (36)	91 (31)

**Carbohydrate, g/day (% daily Energy)**	34 (12)	74 (25)

**Fat, g/day** **(% daily Energy)**	63 (52)	58 (44)

**Mean Kcal/die of the two phases of KEMEPHY**	**1146 ± 88.8**	

Kcal values are expressed in mean per day and SD. Other values are expressed in mean per day.

**Table 2:** Plant extracts used in KEMEPHY (ketogenic Mediterranean with phytoextracts) diet

Plant extracts	*Week 1-3*	*Week 4-6*	*Composition*
Extracts A, ml/day	20	20	Durvillea antarctica, black radish, mint, liquorice, artichoke, horsetail, burdock, dandelion, rhubarb, gentian, lemon balm, chinaroot, juniper, spear grass, elder, fucus, anise, parsley, bearberry, horehound

Extracts B, ml/day	20	20	Serenoa, Red clover, Chervil, Bean, Elder, Dandelion, Uncaria, Equisetum, Horehound, Rosemary

Extracts C, ml/day	50	50	Horsetail, asparagus, birch, cypress, couch grass, corn, dandelion, grape, fennel, elder, rosehip, anise

Extracts D, ml/day (only weeks 1 and 2)	40	0	Eleuthero, eurycoma longifolia, ginseng, corn, miura puama, grape, guaranà, arabic coffee, ginger

The permitted foods were: cooked or raw green vegetables (200 g/meal), meat, fish and eggs (2 times/day), olive oil 40 g/day. Integration with a dish (PAT^® ^i.e. porzione alimentare tisanoreica = tisanoreica nutritional portion) composed of high quality proteins (equivalent to 18 grams) and virtually zero carbohydrate (but that mimic their taste) was provided for every meal, for a maximum of four PATs per day. During the last three weeks complex carbohydrates were introduced (50-80 g/day), cheese (60 g/day), PAT was reduced from four to two, while the other indications remained unchanged. The distribution of nutrients (proteins, carbohydrates and fats) in terms of percentage of total caloric intake was 36%, 12% and 52%, respectively (weeks 1 to 3) and 31%, 25% and 44% (weeks 4 to 6). During the 6 weeks, the patients in the study group consumed 20 ml of extract A, 20 ml of extract B and 50 ml of extract C. During the first two weeks, before breakfast and lunch, they also consumed 40 ml of extract D (Tables 2 and 3).

**Table 3:** Main actives ingredients of used phytoextracts, their reported beneficial effects and related references

Extract	Main Active ingredients	Reported beneficial effects	Refs
A	Mint black radish burdock	- indigestion - antioxidant - choleretic, increases bile secretion helping digestion	[69] [70]

B	Serenoa Repens (saw palmetto)	hormonal regulating effects	[71]

	White bean	alpha-amylase inhibitory properties and has been reported to aid weight loss and glycemic control	[72] [73]

C	Equisetum	Antioxidant diuretic glycemic control	[74] [75]

	Dandelion (Taraxacum officinale	diuretic	[76]

D	Ginseng Miura Puama Guaranà	Ameliorate the commonly reported symptoms of weakness and tiredness during the 1^st ^phase of ketosis (1/2 weeks)	[77] [78] [79]

### Supplements

Subjects also took a daily (1 caplet each morning) multivitamin supplement [23] (containing Magnesium19 mg, Calcium 16 mg, Phosphorus 8 mg, Zinc 4.5 mg, Iron 4.62 mg, Manganese 1 mg, Potassium 0.5 mg, Copper 0.4 mg, Chromium 28.55 μg, Selenium 4 μg, Niacin 10 mg, Beta carotene 1.8 mg, Folic Acid 66 μg, Biotin 30 μg, Vitamin C 19.8 mg, Vitamin E 3.3 mg, Pantothenic Acid 1.98 mg, Vitamin B6 0.66 mg, Vitamin B2 0.53 mg, Vitamin B1 0.426 mg, Vitamin D3 1.65 μg, Vitamin B12 0.33 μg (Multivitaminico Balestra e Mech, Gianluca Mech SpA, Orgiano VI).

### Measurements

Subjects were weighed at the same time of day at the start and after 6 weeks of the diet, using the same weighing scales (Digital Scale Joycare^® ^Jc431). Fasting venous blood samples were collected at weeks 0 and 6 for total cholesterol (CHOLt), triacyglicerol (TG) high-density lipoprotein cholesterol (HDLc), low-density lipoprotein cholesterol (LDLc), glucose, blood urea nitrogen (BUN), uricemia, VES, creatinine, ALT, AST, GGT. Blood was collected in EDTA treated vacutainer tubes. To avoid interassay variation all blood samples were stored at -80° and analysed together at the end of the study. A separate sample of blood was clotted and serum analyzed for total cholesterol and triacylglycerols by photometric assay with an intra-assay and interassay CV values of 2% and 4%, respectively. 

**Figure 1:**
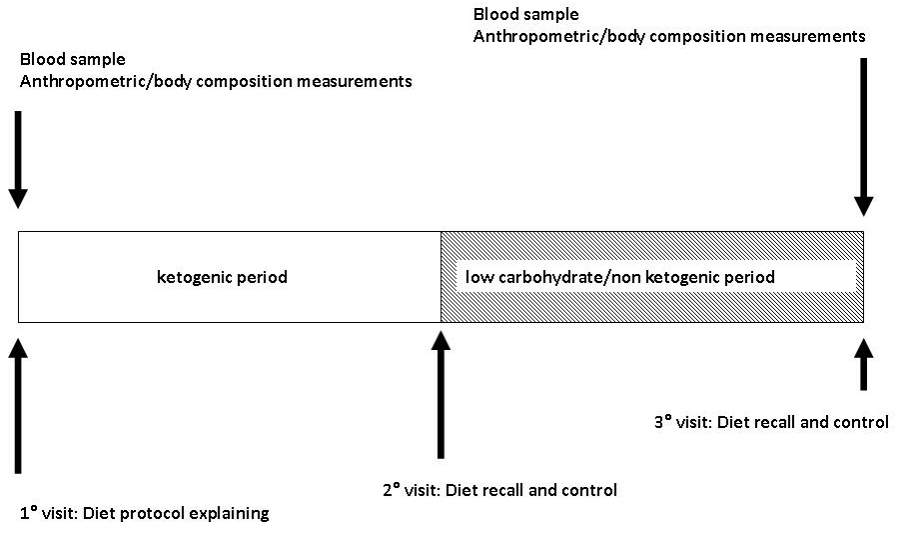
**Experimental design**.

HDL cholesterol was determined using a homogenous enzyme immunoassay. The intra-assay and interassay CV values were both < 4%. Plasma glucose was determined colorimetrically using glucose oxidase methodology. Plasma urea nitrogen were measured using an enzymatic (urease), colorimetric method. Creatinine was measured colorimetrically using the picric acid assay, and uric acid was determined using a modified Trinder peroxide assay. LDLc fraction was calculated from Friedewald's formula: LDLc = TC - HDLc - (TG/5) [24]. Body composition was assessed using bioelectrical impedance analysis (BIA Akern Bioresearch, Pontassieve, FI, Italy) which is a non-invasive and portable method for the estimation of fluid compartments, fat and fat-free mass in healthy subjects. Bioelectrical impedance analysis was chosen for the analysis of body composition due to its reliability, safety, convenience and non-invasiveness making it optimal for the routine monitoring of body composition during the ketogenic diet [25,26]. The experimental design is showed in Figure 1.

### Statistical analysis

We tested the normality and the assumption of homoscedasticity of all parameters at the start of the trial using the Kolmogorov-Sminorv and Shapiro-Wilk tests. To study changes over time and the effects of gender, we added an appropriate interaction analysis. As there were no significant gender interactions, the data of each group were pooled and analyzed together. A Student's t test was used to compare parameters before and after 6 weeks of the KEMEPHY diet using Statistica Software, ver. 8.0 (Tulsa, USA) and the software package GraphPad Prism version 4.00 for Windows, GraphPad Software, San Diego California USA. All data are expressed as mean ± standard deviation.

## Results

Of the 106 persons recruited 87 completed the study (83, 6% compliance). Of the 19 subjects that withdrew 4 were for family/personal reasons, 8 subjects were lost to follow up and only 7 withdrew due to inadequate compliance to the diet protocol. Adjusted for causes of withdrawal only 7 of 104 were not able to follow the KEMEPHY diet for an adjusted compliance of 93.4%

Blood analysis did not reveal significant modification in ALT, AST, GGT and bilirubin values, nor were there significant variations in creatinine, uric acid, nitrogen and electrolytes (NA, K, Cl, Ca, Mg). There were significant changes in lipid profiles with reductions in triglycerides, total cholesterol and LDL along with a rise in HDL levels which all reached significance - see Table 4 and 5.

**Table 4:** Blood biomarker, anthropometric and body composition values, separated for male and female, before and after 6 weeks KEMEPHY diet. Values are expressed in mean and SD.

	Pre diet female	Post diet female	Pre diet male	Post diet male
**BUN (mg/dl)**	33.4 ± 9.5	33.2 ± 8.7	35.2 ± 6.0	33.8 ± 7.0

**Uric acid (mg/dl)**	5.0 ± 1.3	5 ± 1.2	4.8 ± 1.3	5.0 ± 1.3

**VES (mm/hour)**	14 ± 7.3	12.7 ± 7.6	16 ± 7.3	14.4 ± 6.3

**Creatinine (mg/dL)**	0.84 ± 0.15	0.77 ± 0.11	0.83 ± 0.2	0.85 ± 0.2

**Total Cholesterol (mg/dl)**	206.2 ± 41.4	182.8 ± 34.3	199.2 ± 29.8	176.9 ± 26.1

**HDLc (mg/dl)**	46.7 ± 7.2	52.6 ± 9.5	43.9 ± 8.5	50.1 ± 9.1

**LDLc (mg/dl)**	151.8 ± 28.3	137.1 ± 24.8	140.9 ± 32.9	130.4 ± 25.9

**TG (mg/dl)**	119.9 ± 60.3	94.2 ± 41.8	114.1 ± 61.8	93.9 ± 46.2

**ALT (U/l)**	20.5 ± 10.9	17.3 ± 5.1	18.4 ± 4.6	19.1 ± 6.8

**AST (U/l)**	18.5 ± 5.4	17.1 ± 4.7	17.8 ± 3.8	17 ± 5.1

**GGT (U/l)**	20.5 ± 10.9	17.3 ± 5.1	21.5 ± 11.7	15.4 ± 4.1

**Glucose (mg/dl)**	95.7 ± 12.5	90.5 ± 9.8	95.9 ± 11.2	90.6 ± 8

**Weight Kg**	82.6 ± 12.7	76.3 ± 12.1	102.4 ± 22.2	93.4 ± 21

**BMI (Kg/m^2^)**	31 ± 4.8	28.7 ± 4.6	33.6 ± 6.2	30.6 ± 5.8

**% Fat**	42.3 ± 6, 8	36 ± 6, 9	37 ± 4.3	30.6 ± 4.1

**Waist circumference cm**	103.5 ± 14	94, 3 ± 10.3	120.8 ± 15.1	109.7 ± 14.1

**Hip circumference cm**	114.9 ± 11.6	107.2 ± 10.5	117.3 ± 9.9	111.2 ± 10.4

**Table 5:** Blood biomarker values (all subjects) before and after the 6 week KEMEPHY diet. Values are expressed in mean and SD.

	Pre KEMEPHY diet	Post KEMEPHY diets	p
**BUN (mg/dl)**	33.8 ± 8.9	33.4 ± 8.4	n.s.

**Uric acid (mg/dl)**	4.9 ± 1.3	5 ± 1.2	n.s.

**VES (mm/hour)**	14.2 ± 7.2	12.8 ± 7.4	n.s.

**Creatinine (mg/dL)**	0.83 ± 0.16	0.78 ± 0.13	n.s.

**Total Cholesterol (mg/dl)**	204.2 ± 40	181.1 ± 33.4	P < 0.0001

**HDLc (mg/dl)**	46.2 ± 7.4	52.1 ± 7.4	P < 0.0001

**LDLc (mg/dl)**	149.7 ± 29.1	135.8 ± 24.8	P < 0.0001

**TG (mg/dl)**	118.6 ± 59.9	93.8 ± 42.2	P < 0.0001

**ALT (U/l)**	20.7 ± 9.1	18.2 ± 6.7	n.s.

**AST (U/l)**	18.4 ± 5.1	17 ± 5	n.s.

**GGT (U/l)**	21 ± 11	17 ± 5	n.s.

**Glucose (mg/dl)**	96 ± 12	91 ± 9	P < 0.0001

Anthropometric and body composition measurements revealed an average weight loss of 6.72 kg or 7.8% (pre 86.15 ± 16.38 vs post 79.43 ± 15.31; p < 0.0001). Fat mass was reduced from 41.24 ± 6.74 to 34.99 ± 6.74, a fall of 15.1% (p < 0.0001). As in previous studies no significant differences were observed in total body water expressed as percent of body weight [22,27]. Waist circumference was reduced by an average of 9.46 cm from 106.56 ± 15.38 to 97.10 ± 12.69, a fall of 8.9% (p < 0.0001). There were also significant reductions in hip and thigh circumferences of 7.41 cm (from 115 ± 11.24 to 107.78 ± 10.39; p < 0.0001) and 3.32 cm respectively (from 58.65 ± 5.43 to 55.32 ± 4.90). Anthropometric and body composition results are showed in tables 6.

**Table 6:** Anthropometric and body composition measures (all subjects) pre and post diet

	Pre KEMEPHY diet	Post KEMEPHY diets	p
**BMI (Kg/m^2^)**	31.5 ± 5.1	29. ± 4.8	P < 0.0001

**Weight Kg**	86.2 ± 16.4	79.4 ± 15.3	P < 0.0001

**% Fat**	41.2 ± 6.7	35 ± 6.7	P < 0.0001

**Waist circumference cm**	106.6 ± 15.4	97.1 ± 12.7	P < 0.0001

**Hip circumference cm**	115 ± 11.3	107.8 ± 10.4	P < 0.0001

## Discussion

Many weight loss diet procedures continue to focus on the reduction of fat content and controlled protein intake, however our results appear to demonstrate that the KEMEPHY diet, which is a modification of the VLCKD, is effective not only for weight and fat loss but also leads to improvements in the values of various biomarkers which are associated with increased risk of metabolic and cardiovascular disease. The weight loss effect of VLCKD diets may be caused by several factors:

1. Satiety effect of proteins leading to appetite reduction [28-36] in which also ketone bodies may have a role [37,38], although the mechanism is not clear [39];

2. Reduction in lipid synthesis and increased lipolysis mechanisms [40-44];

3. Reduction in at rest respiratory quotient and therefore an increase in fat metabolism for energy use [22,45];

4. Increased metabolic expenditure caused by gluconeogenesis and the thermic effect of proteins [46-51]

The beneficial effects on cardiovascular risk factors involve the reduction of blood triglycerides [17,18,22] and also the reduction of total and LDL cholesterol along with a rise in HDL cholesterol [17,18,22,52-54]. Furthermore the VLCKD can cause modifications in LDL-C particles leading to increased size [53] which may reduce cardiovascular risk since smaller LDL particles have been shown to be more atherogenic [55].

The cholesterol lowering effect of VLCKD is also mediated by the well known facilitating action of insulin on HMGCoA reductase and inhibition of the latter by cholesterol and fats [56]. Insulin then increases the production of endogenous cholesterol while exogenous cholesterol has the opposite effect [56].

The KEMEPHY diet protocol used in this study maintains some advantages of the Mediterranean diet such as the use of olive oil [21] and some vegetables [19] (selected to avoid stimulating insulin production) but at the same time by inducing a physiological ketosis [57] promotes beneficial modifications in cardiovascular risk factors and body composition [22]. The use of the phyoextracts in this study may have contributed to the absence of commonly reported mild effects of ketosis (e.g. weakness, constipation, bad breath, headache). During the first three weeks of the KEMEPHY diet subjects avoided fructose completely and during the second three weeks only a moderate amount of fructose, exclusively from fruit, and therefore together with starch, was permitted. As a matter of fact fructose may stimulate fat biosynthesis via mechanisms which are not yet fully characterised [58], also several studies have reported that excessive concentrations of fructose can induce some or all of the features of metabolic syndrome independently of energy intake. Clinical and epidemiologic data further suggest that excessive fructose intake can contribute to the causes of metabolic syndrome [59].

Adjusted compliance in this study was 93.4% which is higher than reported compliances of standard VLCK diets (in the 20% to 58% range). [22,60,61]. It is tempting to speculate that the inclusion of "carbohydrate-like" formulated foods is one of the reasons for high compliance - however this, along with the potential benefits of the phytoextracts, requires further verification in a future study with a matched control group.

### Safety considerations

If we assimilate *de facto*, which is not always correct, ketogenic diets with high protein diets then the risks proposed by critics of this type of dietary approach are essentially those of possible kidney damage due to high levels of nitrogen excretion during protein metabolism which can cause an increase in glomerular pressure and hyper-filtration [27]. There is not wide agreement between studies however, some infer the possibility of renal damage from animal studies [62,63] while others, looking at both animal models and human studies propose that even high levels of protein in the diet do not damage renal function [64,65]. In subjects with intact renal function higher dietary protein levels caused some functional and morphological adaptations without negative effects [66]. Also it should be underlined that ketogenic diets are only *relatively *high in protein [49,67] and that some recent studies have demonstrated that VLCKD can even cause a regression of diabetic nephropathy in mice [68]. With regard to possible acidosis during VLCKD since the concentration of ketone bodies never rises above 8 mmol/l [40,42] this risk is virtually inexistent in subjects with healthy insulin function.

## Conclusions

Some limitations to this study include the lack of a matched control group and the short trial period. There are though many studies that demonstrate that VLCK diets are more effective than low fat or standard Mediterranean diets, at least over the short term [21], and the main aims of the present initial study were to assess safety aspects, acceptance & palatability and weight loss & biomarker changes. We are able to conclude that at least in the short term it was able to lead to positive changes including the reduction of fasting blood glucose, improvements in lipid profiles, significant and rapid weight and fat loss with the preservation of lean mass. We also note a high level of compliance, whether this was due to specific unique features of the present diet requires confirmation in a future matched control trial.

## Abbreviations

KEMEPHY: ketogenic Mediterranean with phytoextracts; VLCKD: very low carbohydrate ketogenic diet; high-density lipoprotein cholesterol; LDLc: low-density lipoprotein cholesterol; TG: Triglycerides; GLU: Glucose; BUN: Blood Urea Nitrogen; UA: Uric acid; ALT: Alanine aminotransferase; AST: Aspartate Aminotransferase; GGT Gamma-glutamyl transpeptidase; Cr: Creatinine.

## Competing interests

This work was partially funded by Gianluca Mech SpA, Orgiano (VI), Italy. AP and LC research activity is funded by dept. of Human Anatomy and Physiology, University of Padova; KG research activity is funded by the Biomedical Engineering Laboratory, Institute of Communication and Computer Systems, National Technical University of Athens, Athens, Greece.

LC is scientific consultant for Gianluca Mech SpA, Orgiano (VI), Italy.

Investigators conducted the study in its entirety and maintained exclusive control of all data and analyses. The funding source had no involvement in any part of the recruitment of participants, study intervention, data collection, data analyses, interpretation of the data, or preparation or review of this manuscript.

## Authors' contributions

AP was the main researcher and was responsible for study design, statistical analysis and interpretation of data and draft of manuscript. conceived the study, participated in its design, drafted the manuscript and performed the statistical analysis. LC was responsible for study design and acquisition of data. KG was was responsible for analysis and interpretation of data and helped to draft the manuscript. All authors read and approved the final manuscript.
